# Impact of TR_34_/L98H, TR_46_/Y121F/T289A and TR_53_ Alterations in Azole-Resistant *Aspergillus fumigatus* on Sterol Composition and Modifications after In Vitro Exposure to Itraconazole and Voriconazole

**DOI:** 10.3390/microorganisms10010104

**Published:** 2022-01-04

**Authors:** Rose-Anne Lavergne, Marjorie Albassier, Jean-Benoît Hardouin, Carlos Alvarez-Moreno, Fabrice Pagniez, Florent Morio, Patrice Le Pape, Isabelle Ourliac-Garnier

**Affiliations:** 1 Nantes Université, CHU de Nantes, Cibles et Médicaments des Infections et de l’Immunité, IICiMed, UR 1155, F-44000 Nantes, France; Rose-Anne.Lavergne@univ-nantes.fr (R.-A.L.); Florent.Morio@univ-nantes.fr (F.M.); Patrice.Le-Pape@univ-nantes.fr (P.L.P.); 2 Nantes Université, Cibles et Médicaments des Infections et de l’Immunité, IICiMed, UR 1155, F-44000 Nantes, France; Marjorie.Albassier@univ-nantes.fr (M.A.); Fabrice.Pagniez@univ-nantes.fr (F.P.); 3 Nantes Université, Univ Tours, INSERM, Methods in Patients-Centered Outcomes and Health Research, SPHERE, UMR 1246, F-44000 Nantes, France; jean-benoit.hardouin@univ-nantes.fr; 4 Service de Santé Publique et Plateforme de Méthodologie et Biostatistique, CHU de Nantes, F-44000 Nantes, France; 5 Departamento de Medicina Interna, Facultad de Medicina, Universidad Nacional de Colombia, Bogota 111176, Colombia; caalvarezmo@unal.edu.co; 6 Clínica Colsanitas Groupo Keralty, Clínica Universitaria Colombia, Bogotá 111176, Colombia

**Keywords:** *Aspergillus fumigatus*, sterol, ergosterol, azole-resistance, tandem repeat

## Abstract

Background: Sterols are the main components of fungal membranes. Inhibiting their biosynthesis is the mode of action of azole antifungal drugs that are widely used to treat fungal disease including aspergillosis. Azole resistance has emerged as a matter of concern but little is known about sterols biosynthesis in azole resistant *Aspergillus fumigatus*. Methods: We explored the sterol composition of 12 *A. fumigatus* isolates, including nine azole resistant isolates with TR_34_/L98H, TR_46_/Y121F/T289A or TR_53_ alterations in the *cyp51A* gene and its promoter conferring azole resistance. Modifications in sterol composition were also investigated after exposure to two azole drugs, itraconazole and voriconazole. Results: Overall, under basal conditions, sterol compositions were qualitatively equivalent, whatever the alterations in the target of azole drugs with ergosterol as the main sterol detected. Azole exposure reduced ergosterol composition and the qualitative composition of sterols was similar in both susceptible and resistant isolates. Interestingly TR_53_ strains behaved differently than other strains. Conclusions: Elucidating sterol composition in azole-susceptible and resistant isolates is of interest for a better understanding of the mechanism of action of these drugs and the mechanism of resistance of fungi.

## 1. Introduction

Invasive aspergillosis is a life-threatening infection in immunocompromised patients mainly due to *Aspergillus fumigatus*. Azole-drugs (itraconazole, voriconazole, posaconazole and isavuconazole) are the first-line therapy for prevention and treatment of *Aspergillus* infections [1,2]. They act by inhibiting the 14α-lanosterol demethylase, which has a key role in ergosterol biosynthesis [3]. Ergosterol is an important component of fungi, involved in membrane fluidity and cell integrity [4]. The burden of azole resistance in *A. fumigatus* has been pointed out since 2007 in Europe [5,6]. This worrisome problem has spread worldwide [7]. Mechanism of azole-resistance in *A. fumigatus* is mainly due to alteration in the *cyp51a* gene and/or its promotor, encoding for the 14α-demethylase. Two routes of azole-resistance are currently admitted: (i) the “clinical route” of azole resistance, in long-term treated patients with azole drugs and (ii) the “environmental route” of azole resistance, due to the wide use of azole fungicides in agriculture called demethylation inhibitors (DMI) which chemical structures are very close to clinical azole drugs [8]. Until now, the clinical route of azole resistance was associated with point mutations in the *cyp51a* gene while the environmental route of azole resistance led to complex alteration with tandem replication of a part of the promoter of the *cyp51a* gene, with or without point mutations in the *cyp51a* gene (TR_34_/L98H, TR_46_/Y121F/T289A, TR_53_…) [9].

As ergosterol biosynthesis is targeted by azole compounds, elucidating sterol composition in azole-susceptible and resistant *A. fumigatus* is of interest to understand the mechanism of action of these drugs and the mechanism of resistance of fungi. The ergosterol biosynthesis pathway has been described for some fungal pathogens such as *Candida albicans* [10], *Cryptococcus neoformans* [11], *A. fumigatus* [12] and more recently for *Mucorales* [13]. Concerning *A. fumigatus*, sterol studies mostly focused on azole-susceptible clinical isolates. So far, a single itraconazole-susceptible environmental strain has been studied [14]. In the few azole-resistant strains studied, resistance was induced by exposure to azole compounds [15] or by engineered mutations [12,16,17,18]; only one study included azole-resistant isolates from clinical origin [12]. Under basal condition, whatever the mutation in *cyp51A*, ergosterol remained the main sterol in azole-sensitive and azole-resistant strains and only minor changes in sterol profiles were observed [12,14]. Mutations in other genes encoding enzymes involved in ergosterol biosynthesis (*erg3A, erg3B* and *hmg1*) [16,17] or in the negative cofactor 2 complex (a transcriptional regulator) [18] modified the total ergosterol content and relative distribution of sterols. Some results of these studies are presented in Section 3.2.1. To our knowledge, the effect of azole drugs on ergosterol biosynthesis was studied only once, with voriconazole and isavuconazole [19].

The aims of this study were (i) to describe the sterol composition of azole resistant isolates harbouring environmental alterations (TR_34_/L98H, TR_46_/Y121F/T289A and TR_53_) and (ii) to explore how exposure to azole drugs, namely itraconazole and voriconazole, could affect their sterol composition in comparison with azole-susceptible isolates.

## 2. Materials and Methods

### 2.1. Isolates of A. fumigatus

Twelve isolates, gathered in four groups of three isolates, were explored during this study: azole-susceptible isolates (Group 1) and azole-resistant isolates, bearing TR_34_/L98H (Group 2) or TR_46_/Y121F/T289A (Group 3) or TR_53_ (Group 4) alterations. Itraconazole and voriconazole MICs were determined according to the EUCAST reference method [20]. When EUCAST MIC was >8 mg/L or >16 mg/L for itraconazole or voriconazole, respectively, the Etest^®^ method (bioMerieux, Marcy l’Etoile) was additionally done to be closest to the MIC (Etest^®^ strips concentration gradient up to 32 µg/mL). Characteristics of all isolates are given in Table 1.

### 2.2. Culture Conditions

A 4 × 10^7^ spores/mL suspension in sterile water with Tween 20 (0.1%) was prepared from a fresh culture. Two hundred microliters were then inoculated to 25 mL of YPD (Yeast Peptone Dextrose) 20% glucose broth. Either antifungal agent (itraconazole and voriconazole, stock solution at 1.6 mg/mL in DMSO) or DMSO (basal condition) was then added to obtain 0.25 × MIC or 32 mg/L for strains with MIC >32 mg/L (Table 1). Cultures were incubated for 48 h at 37 °C under agitation (130 rpm). Each experiment was repeated three times. Absence of sterols in YPD broth was verified before running these experiments.

### 2.3. Total Sterol Extraction

Mycelia obtained from 48 h incubation were harvested by filtration on two layers of Whatman filter paper (n°1), washed with distilled water and weighted. The first step of sterol extraction was saponification, with the addition of 5 mL of fresh ethanolic potassium hydroxide solution, and incubation in water bath during 90 min at 90 °C. The non-saponifiable lipids (sterols) were extracted twice by adding 2 mL of hexane and manual mixing. Once the two layers were separated, the upper layer was harvested in a new tube. The organic layer was then washed twice by adding 1 mL of sterile water to the 4 mL of hexane. Organic phase was then dried by adding sodium sulfate. Final organic phase was transferred to a new collection tube after a 5 min centrifugation step at 720 g.

### 2.4. Sterol Derivatization

A volume of extract equivalent to 100 mg mycelia was transferred into a glass tube and hexane was evaporated by heating at 80 °C. Cholesterol was added as internal standard (2.5 µg). Sterols were derivatized with 100 μL of *N*-Méthyl-*N*-triméthyl-silyltrifluoroacetamide (TMS) (Sigma-Aldrich, Saint-Quentin-Fallavier, France) during 30 min at room temperature. The solvent was then evaporated at 80 °C under air flux. Dried sterols were solubilized with 200 μL dichloromethane and stored at −20 °C until analysis.

### 2.5. Sterol Content Analysis

Sterols as TMS derivatives were analyzed by Gas Chromatography-Mass Spectrometry (GC-MS) using an Agilent 7890A GC system, with a HP-5MS column (60 m × 0.25 mm, 0.25 μm, Agilent, Les Ulis, France) coupled with a mass detector (Agilent 5975C inert MSD—E.I. 70 eV). One microliter of each sample was injected in splitless mode at 250 °C. The carrier gas was helium at a flow rate of 1.2 mL/min. The oven was set at 150 °C for 0.5 min and then raised to 290 °C at 50 °C/min and from 290 °C to 305 °C at 2 °C/min for 7 min then 10 °C/min to 315 °C for 16 min. Sterols were identified via their electron ionization fragmentation pattern by comparison of the mass spectrum of each isolated sterol with previously published spectra (AMDIS data bank, [21,22,23]). Cholesterol was used as internal standard to calculate relative retention time. Sterol composition was expressed as a relative amount, i.e., percentage of the total amount of sterols detected.

For comparison between isolates and/or between conditions, only sterols with an amount of 1% or more were considered. Values are expressed as the mean of three independent experiments. Percentages lower than 1% have been obtained mathematically when this sterol has been detected only one or two times with a value higher than 1%. Relative amount and qualitative analysis of sterols were done using MSD ChemStation Data Analysis Application and AMDIS software.

### 2.6. Statistical Analysis

Univariate analyses of ergosterol content were performed with non-parametric two-tailed tests (Kruskal Wallis and Mann Whitney tests) with a type-I error fixed to 5%. Multivariate analyses were performed using ANOVA. Statistical analysis was performed using GraphPad Prism 7 and Stata 17 software.

## 3. Results and Discussion

### 3.1. Identification of Sterols

**Figure 1:**
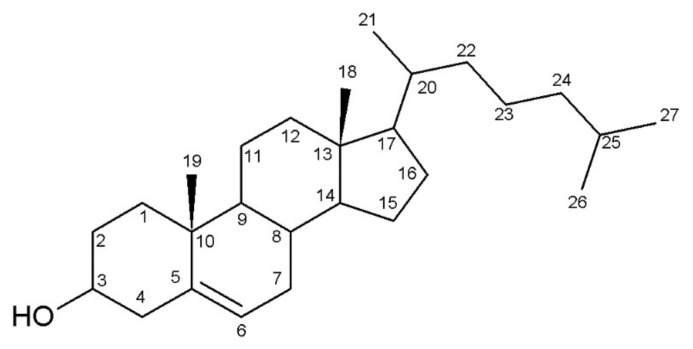
Basic structure of a sterol with standard carbon numbering according to the IUPAC [30].

Thirteen sterols have been detected during this study and are listed in Table 2. Nomenclature of numbering sterols is presented in Figure 1. Eleven sterols have been identified and two are referred as “Unknown 1” and “Unknown 2” (PM 480 and PM 470 respectively). Ergosterol E (ergosta-5,7,22E-trien-3ß-ol) is the main sterol of the fungal membrane. Two ergosterol isomers (ergosta-5,7,22E-trien-3ß-ol and ergosta-5,7,22Z-trien-3ß-ol) were also identified but ergosterol Z has never been described in previous fungal sterol analysis. We hypothesized that isomerization could have occurred due to cell culture conditions and these two isomers’ quantifications have been pooled in all the analysis and considered as “ergosterol”.

**Figure 2:**
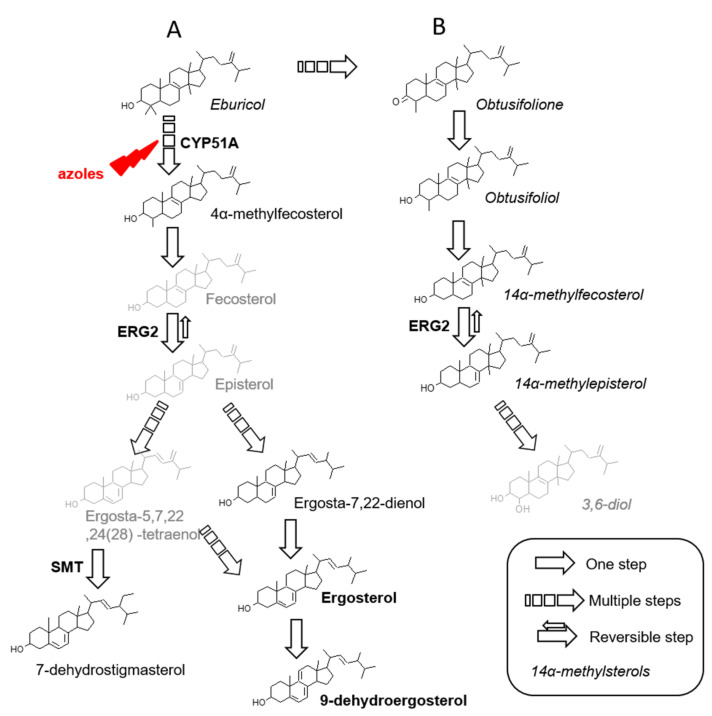
*Aspergillus fumigatus* sterol biosynthesis pathway (**A**) Possible pathway for ergosterol biosynthesis (**B**) Alternative pathway, after CYP51 inhibition. In grey sterol intermediates not detected in this study. SMT: sterol methyltransferase.

Overall, five sterols bearing a methyl in C14 position (14α-methyl sterols) have been identified: eburicol, obtusifoliol, obtusifolione, 14α-methylfecosterol and 14α-methylepisterol. Whereas eburicol is upstream in the biosynthesis pathway of ergosterol, the remaining four belong to an alternative pathway that ends into the synthesis of potentially toxic sterols (Figure 2). In our chromatographic conditions, because of the poor separation of obtusifoliol and obtusifolione when both present in a sample, we quantified them together.

In comparison to vertebrates, sterols from higher plants and fungi differ by the alkylation of the side chain at C24 position [31]. In fungi, most of C24 alkylated sterols are methylated (lanosterol, eburicol, obtusifoliol), but an ethylation is also possible as observed in higher plants, depending on the activity of S-adenosyl-L-methionine sterol C24 methyl transferases (SMTs). These enzymes have been studied in phytosterols biosynthesis and in *Saccharomyces cerevisiae* [32] but C24-ethylsterols and SMT are unexplored in *A. fumigatus* [4]. This sterol could be produced in two steps from 24-methylcholesta-5,7,24(28)-trien-3β-ol involving Erg6 (first step) then Erg4 and Erg5 (second step), as proposed by Bouvier-Navé for the formation of 4,4,14α-trimethyl-5α-stigmasta-8,22-dien-3β-ol from eburicol [31]. Here, we observed a C24 alkylated sterol, 7-dehydrostigmasterol (7-DHS: 24-éthylcholesta-5,7,22-trien-3β-ol) for all the isolates (Figure 2). In protozoans, such as *T. cruzi* and *Leishmania* spp. the major sterol components are ergosterol and its 24-alkylated and methylated derivatives i.e., 7-DHS [33]. Concerning the three last sterols identified, ergosta-7,22-dienol and 4α-methyl-fecosterol are common intermediates in *A. fumigatus* ergosterol biosynthesis pathway whereas 9-dehydroergosterol is the result of ergosterol degradation (Figure 2).

### 3.2. Qualitative Composition and Relative Amount of Sterols

Sterol membrane content determined for each strain in each experimental condition are presented in Table 3, Table 4, Table 5 and Table 6. Strains have been classified in four groups: itraconazole and voriconazole susceptible isolates (Group 1, Table 3), resistant isolates with TR_34_/L98H (Group 2, Table 4) or TR_46_/Y121F/T289A (Group 3, Table 5) or TR_53_ (Group 4, Table 6) alterations. Whatever the mutations in *cyp51A* gene, the alterations in its promoter and the growth conditions, ergosterol was the main sterol detected, accounting from 79.5% to 89.5% under basal conditions and from 61.8% to 81.1% and from 44.4% to 74.2% when exposed to itraconazole or voriconazole, respectively.

#### 3.2.1. Under Basal Conditions

Under basal conditions besides ergosterol, 7-DHS (a C24-ethylsterol) was always detected, accounting from 10.5% to 20.0%, whatever the alteration involved. Sterols, other than ergosterol and 7-DHS, were inconstantly detected in only three isolates: obtusifoliol, obtusifolione and eburicol for AF1861 (susceptible strain), 4-methylfecosterol for AF23 (TR_34_/L98H strain) and 9-dehydroergosterol for AF1897 (TR_34_/L98H strain). This last sterol probably results from the degradation of ergosterol or 5,7-diene sterols [12]. Table 7 sums up the sterol composition of six wild type strains of *A. fumigatus* previously published in four articles between 2001 and 2019 [12,13,14,16,19]. Only sterols observed in our strains in basal conditions were detailed here i.e., ergosterol, ergosta-7,22-dienol, 7-DHS and eburicol. Differences in sterol content can be influenced by conditions of culture [34], by strains themselves and also by chromatographic conditions that have evolved along years. Ergosterol was always the main sterol with a variable amount (from 73.5 to 95%) and eburicol was also detected (from 0.3 to 2.72%). 7-DHS was only observed three times with an amount varying from 0.9 to 19.4% (in an environmental strain). Ergosta-7,22-dienol was only observed in CM237 strain by Alcazar-Fuoli et al. Interestingly 9-dehydroergosterol, 4-methylfecosterol, obtusifoliol and obtusifolione had never been reported. In *Candida albicans*, the balance of ergosterol and 14α-methylsterols is controlled by NSG2, a protein containing INSIG domain (insulin-induced genes) [35]. It would be interesting to search for putative orthologous genes to NSG2 in AF1861 isolate to explain the basal detection of obtusifoliol and obtusifolione.

#### 3.2.2. Under Itraconazole or Voriconazole Exposure

When exposed to itraconazole or voriconazole, alongside the accumulation of eburicol, appearance of 14α-methylsterols (obtusifoliol, obtusifolione, 14α-methylfecosterol and 14α-methylepisterol) was observed for all strains, except for AF1861, where obtusifoliol and obtusifolione only increase as these sterols were detected in low amount in basal condition. These observations are linked to the mechanism of action of azole drugs (inhibitors of the 14α-demethylase), which lead to the accumulation of 14α-demethylase substrates and trigger deviation to a new biosynthesis pathway from eburicol and leading to synthesis of toxic sterol (3,6-diol) or at least its precursors [36] (Figure 2). Obtusifolione and obtusifoliol have been described in *C. albicans* after exposure to various azole drugs [37,38]. After ketoconazole (imidazole drug targeting 14α-demethylase) treatment, *A. fumigatus* accumulated obtusifoliol, 14α-methylfecosterol and 14α-methyl-ergosta-5,7,22,24(28)-tetraene-3βol [39]; obtusifoliol was also observed after triarimol (pyrimidines fungicide inhibiting 14α-demethylase) treatment [40]. In our study, we observed 14α-methylfecosterol only once but 14α-methylepisterol was constantly detected after voriconazole treatment and sometimes after itraconazole treatment. Conversion of fecosterol to episterol implies ERG2, a C8 isomerase [12]. This step is the unique reversible reaction in the biosynthesis of ergosterol but rather to the benefit of episterol synthesis [12], in ratio of 19:1 as shown in cholesterol biosynthesis pathway [41]. We hypothesized that both 14α-methylfecosterol and 14α-methylepisterol could be produced without the possibility of detection of 14α-methylfecosterol because of its shift toward the formation of 14α-methylepisterol (Figure 2). Ergosta-7,22-dienol, a precursor of ergosterol, was detected in four isolates (AF23 and AF1897 with TR_34_/L98H alteration, AF2226 with TR_46_/Y121F/T289A alteration and AF84 with TR_53_ alteration), when exposed to itraconazole. This intermediate between episterol and ergosterol was detected previously in basal conditions in *A. fumigatus* [12]. Interestingly, in *Candida*, ergosta-7,22-dienol is the ultimate sterol synthetized (instead of ergosterol) when erg3 is mutated but is never detected as intermediate of ergosterol biosynthesis [37]. Finally, after exposure to itraconazole or voriconazole, two sterols remained unidentified, but they were inconstantly present, and in a very low amount.

### 3.3. Statistical Comparison of Ergosterol Content

**Figure 3:**
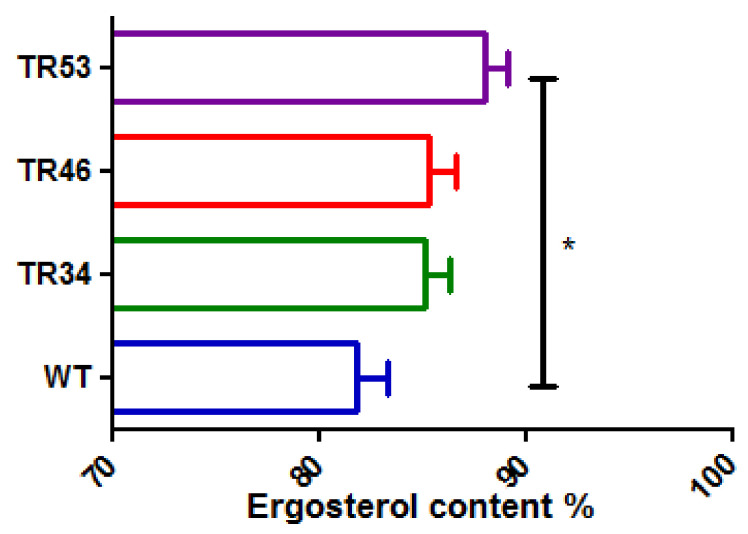
Relative content of ergosterol in basal condition. WT: susceptible isolates, TR34: TR_34_/L98H, TR46: TR_46_/Y121F/T289A. * *p* = 0.006 (Mann Whitney test).

Under basal condition, ergosterol content was not significantly different comparing the three isolates in each group with the same kind of alteration (nonparametric Kruskal Wallis test). There was also no significant difference in ergosterol content between azole-susceptible isolates and azole resistant isolates bearing TR_34_/L98H or TR_46_/Y121F/T289A but it was significantly higher in azole resistant isolates bearing TR_53_ alterations than in azole-susceptible isolates (nonparametric Mann Whitney test, *p* = 0.006) (Figure 3).

When exposed to itraconazole, the same observation was made: ergosterol content was not significantly different in each group of three isolates with the same kind of alteration (nonparametric Kruskal Wallis test), there is no significant difference in ergosterol content between azole-susceptible isolates (WT) and azole-resistant isolates bearing TR_34_/L98H or TR_46_/Y121F/T289A alterations but ergosterol content was lower in azole-resistant isolates bearing TR_53_ alteration than in azole-susceptible isolates (nonparametric Mann Whitney test, *p* = 0.0272). When exposed to voriconazole, ergosterol content was not significantly different in each group of three isolates with the same kind of alteration and ergosterol content was not different comparing each group of isolates with the same alteration to azole-susceptible isolates (nonparametric Kruskal Wallis test).

Then using ANOVA, an analysis of all ergosterol data together (whatever the alteration and the growth condition) was performed. In this model of analysis, ergosterol content was not significantly different for azole-susceptible, TR_34_/L98H and TR_46_/Y121F/T289A groups. The unique difference in ergosterol content concerned TR_53_ isolates (Group 4), when exposed to itraconazole. Overall ergosterol content reach 85.1% (IC 95 [82.8%; 87.4%]) whatever susceptibility or resistance to azoles and whatever growth conditions. This observation, together with the same sterol composition in basal condition suggested that neither TR_34_/L98H, nor TR_46_/Y121F/T289A nor TR_53_ alterations affected biosynthesis of ergosterol. The L98H modification in the 14α-steroldemethylase affect neither the biological activity of this protein nor the access of the natural ligands to the active site [42]; the duplication of 34 bases allows a better binding of cyp51A activator (SrbA) than repressor [43] leading to overexpression of cyp51A mRNA [42]. This knowledge about the mechanisms of resistance mediated by TR_34_/L98H alterations explains our findings on ergosterol content in basal condition. As for TR_34_, TR_46_ is associated with an overexpression of cyp51A mRNA while the Y121F mutation destabilizes the active site of the enzyme [44]. Basal sterols content in the 3 TR_46_/Y121F/T289A strains suggests that the enzyme activity is not affected by these alterations. TR_53_ alteration is also associated with a duplication of SRE1 and SRE2 (Sterol Regulatory Element) allowing a better binding of cyp51A activator (SrbA) than repressor [43]. In our growth conditions, itraconazole treatment decreased ergosterol content by 10.8% (IC 95 [7.3%; 14.3%]) for WT, TR_34_/L98H and TR_46_/Y121F/T289A isolates while ergosterol reduction was more important for TR_53_ isolates than for other groups (18.6%, IC 95 [13.5%; 23.8%]). Voriconazole treatment had the same impact for all isolates, with ergosterol decreasing by 20.7% (IC 95 [17.4%; 23.9%]).

### 3.4. Statistical Comparison of 14α-Methylsterols Content

Under exposure to itraconazole or voriconazole, 14α-methylsterols content was not significantly different in each group of three isolates with the same kind of alteration (nonparametric Kruskal Wallis test). Using ANOVA model, itraconazole exposure had the same effect on WT, TR_34_/L98H and TR_46_/Y121F/T289A isolates: 12.8% (IC 95 [9.4%; 16.1%]) of 14α-methylsterols were quantified. By comparison, TR_53_ isolates contained more 14α-methylsterols after itraconazole exposure, with 22.6% (IC 95 [16.8%; 28.4%]). Whatever the azole-susceptibility or resistance of strains, voriconazole had the same effect on 14α-methylsterols content (ANOVA). Overall, 14α-methylsterols content reach 31.4% (IC 95 [28.5%; 34.3%]) when exposed to voriconazole. Therefore, in our conditions, voriconazole appeared to be a stronger inhibitor of 14α-demethylase than itraconazole. Exposure to itraconazole or voriconazole at a concentration adapted to MIC of each isolate, can explain the same amount of 14α-methylsterols in azole-susceptible and azole-resistant strains with TR_34_/L98H or TR_46_/Y121F/T289A alterations. The TR_53_ genotype is not associated with Cyp51 mutations so that impairing the binding of azoles is not expected, which can explain a more important inhibition of ergosterol biosynthesis than for TR_34_/L98H or TR_46_/Y121F/T289A isolates. Interestingly, no 14-methylergosta-8,24-dien-3,6-diol was detected. It is the ultimate sterol after blockade of the 14α-demethylase and it is known to be toxic through the impairment of the membrane function of sterol due to the presence of the 6- hydroxy group [45,46]. We made the hypothesis that this absence of 14-methylergosta-8,24-dien-3,6-diol may be explained by the concentration of itraconazole or voriconazole added to fungal culture, which is not enough to end into the synthesis of this toxic sterol.

## 4. Conclusions

In this study, we described for the first-time relative sterol composition of azole-resistant *Aspergillus* isolates with complex alterations in the *cyp51A* gene and/or its promotor, which are linked to environmental exposition to azole fungicides. This study highlights that TR_34_/L98H and TR_46_/Y121F/T289A alterations had no impact on relative composition of sterols in basal conditions when compared with susceptible isolates. Moreover, in our conditions, after itraconazole and voriconazole exposure the qualitative composition of sterols was similar in susceptible and resistant isolates. Interestingly in basal condition relative ergosterol content was higher for TR_53_ strains and it was significantly lower than for other alterations after itraconazole and voriconazole treatment. It would be interesting to look for other mechanisms of resistance in these isolates. Moreover, in this study we did not explore point substitution in 14α-lanosterol demethylase (including M220 and G54); it could be of interest to compare the effects of point mutation with the effects of complex alterations studied here.

## Figures and Tables

**Table 1:** Characteristics of isolates.

Name	Origin	CYP51A Alteration	MIC Itraconazole mg/L	MIC Voriconazole mg/L
ATCC 204305	NA		0.25 (S)	0.5 (S)
AF1861	C		0.125 (S)	1 (S)
AF1799	C		0.5 (S)	1 (S)
AF899	C	TR_34_/L98H	>32 (R)	8 (R)
AF1897	C	TR_34_/L98H	>32 (R)	16 (R)
AF23	E	TR_34_/L98H	>32 (R)	16 (R)
AF1168	C	TR_46_/Y121F/T289A	1 (S)	>32 (R)
AF2226	C	TR_46_/Y121F/T289A	8 (R)	>32 (R)
AF1468	C	TR_46_/Y121F/T289A	8 (R)	>32 (R)
AF84	E	TR_53_	>32 (R)	3 (R)
AF112	E	TR_53_	>32 (R)	16 (R)
AF124	E	TR_53_	6 (R)	2 (R)

C: clinical; E: environnemental; S: susceptible; R: resistant; NA: not applicable, reference strain.

**Table 2:** List of the 13 sterols present with an amount higher than 1% of the total amount of sterols and identified during this study.

Identified Sterols	IUPAC Name	MW_(TMS)_	RRT	Reference
9-dehydroergosterol	24-methylcholesta-5,7,9(11),22-tetraen-3ß-ol or ergosta -5,7,9(11),22-tetraen-3ß-ol	466	1.05	[24,25]
Unknown 1		480	1.06	
Ergosterol Z Ergosterol E	24-methylcholesta-5,7,22-trien-3ß-ol or ergosta-5,7,22Z-trien-3ß-ol or ergosta-5,7,22E-trien-3ß-ol	468	1.06 1.10	[26]
Ergosta-7,22-dienol	24-methylcholesta-7,22-dien-3ß-ol or ergosta-7,22-dien-3ß-ol	470	1.13	[24]
14α-methylepisterol	14α,24- dimethylcholesta-7,24(28)-dien-3ß-ol or 14-methylergosta-7,24(28)-dien-3ß-ol	484	1.14	
Unknown 2		470	1.18	
7-dehydrostigmasterol	24 ethyl-cholesta-5,7,22-trienol	482	1.19–1.20	[27]
14α-methylfecosterol	14α,24- dimethylcholesta-8,24(28)-dien-3ß-ol or 14α-methylergosta -8,24(28)-dien-3ß-ol	484	1.20	[28]
Obtusifolione	4α,14α,24-trimethylcholesta-8,24(28)-dien-3ß-one or 4α,14α-dimethylergosta-8,24(28)-dien-3ß-one	424	1.22	[29]
Obtusifoliol	4α,14α,24-trimethylcholesta-8,24(28)-dien-3ß-ol or 4α,14α-dimethylergosta-8,24(28)-dien-3ß-ol	498	1.23	[24]
4-methylfecosterol	4α,24- dimethylcholesta-8,24(28)-dien-3ß-ol or 4α- methylergosta-8,24(28)-dien-3ß-ol	484	1.25	[24]
Eburicol	4α,4ß,14α,24-tetramethylcholesta-8,24(28)-dien-3ß-ol or 4α,4ß,14α-trimethylergosta-8,24(28)-dien-3ß-ol	512	1.31	[28]

MW: Molecular Weight, expressed as TMS derivatives (g/mol), RRT: relative retention time (sterol retention time compared with cholesterol retention time).

**Table 3:** Relative composition of sterols of group 1, azole-susceptible strains, under basal conditions or after in vitro exposure to itraconazole (ITC) or voriconazole (VRC) (concentration = 0.25 × MIC). Values are expressed as the mean of three independent experiments, standard deviation is given in brackets.

	ATCC 204305			AF1861			AF1799		
	Basal	ITC	VRC	Basal	ITC	VRC	Basal	ITC	VRC
ergosterol Z et E	80.0 (±0.4)	81.1 (±5.3)	70.4 (±2.9)	79.5 (±6.1)	78.8 (±4.1)	68.5 (±5.6)	85.9 (±2.7)	68.3 (±56.9)	65.3 (±2.0)
Unknown 1			1.3 (±1.5)						
14α-methylepisterol			1.6 (±0.2)			1.8 (±0.4)		2.4 (±0.5)	3.0 (±0.8)
7-dehydrostigmasterol	20.0 (±0.4)	13.9 (±7.0)	9.9 (±1.1)	18.9 (±5.5)	17.0 (±4.0)	2.1 (±1.0)	14.2 (±2.6)	1.0 (±1.7)	0.7 (±1.2)
obtusifoliol + obtusifolione		2.4 (±2.5)	8.8 (±1.0)	0.8 (±1.4)	1.5 (±0.1)	13.3 (±3.3)		12.6 (±3.1)	14.1 (±0.8)
eburicol		2.5 (±1.3)	8.1 (±1.2)	0.8 (±1.3)	2.8 (±0.5)	14.4 (±1.3)		15.8 (±6.4)	16.9 (±2.1)

**Table 4:** Relative composition of sterols of group 2, strains with TR_34_/L98H alterations, under basal conditions or after in vitro exposure to itraconazole (ITC) (concentration = 32 mg/L) or voriconazole (VRC) (concentration = 0.25 × MIC). Values are expressed as the mean of three independent experiments, standard deviation is given in brackets.

	AF23			AF0899			AF1897		
	Basal	ITC	VRC	Basal	ITC	VRC	Basal	ITC	VRC
9-dehydroergosterol							0.4 (±0.7)		
ergosterol Z et E	82.9 (±2.7)	74.0 (±5.5)	68.8 (±3.4)	88.0 (±1.0)	72.8 (±3.3)	69.5 (±3.1)	84.6 (±4.3)	69.5 (±5.5)	44.4 (±9.7)
ergosta-7,22-dienol		0.3 (±0.6)						0.4 (±0.7)	
14α-methylepisterol			1.1 (±0.2)			1.9 (±0.2)			1.6 (±1.6)
unknown 2						0.5 (±0.9)			
7-dehydrostigmasterol	16.7 (±2.1)	16.4 (±6.8)	5.7 (±1.7)	12.0 (±0.9)	12.8 (±5.0)	4.9 (±3.9)	15.0 (±3.6)	14.6 (±4.4)	0.7 (±1.2)
obtusifoliol + obtusifolione		2.0 (±0.6)	10.4 (±2.8)		4.4 (±0.4)	10.0 (±2.1)		4.4 (±0.6)	20.3 (±4.3)
4-methyl-fecosterol	0.3 (±0.6)								
eburicol		8.0 (±0.4)	16.3 (±5.4)		10.0 (±2.3)	13.2 (±4.6)		11.1 (±2.6)	33.0 (±7.9)

**Table 5:** Relative composition of sterols of group 3, strains with TR_46_/Y121F/298A alterations, under basal conditions or after in vitro exposure to itraconazole (ITC) (concentration = 0.25 × MIC) or voriconazole (VRC) (concentration = MIC). Values are expressed as the mean of three independent experiments, standard deviation is given in brackets.

	AF1168			AF2226			AF1468		
	Basal	ITC	VRC	Basal	ITC	VRC	Basal	ITC	VRC
ergosterol Z et E	82.8 (±2.5)	79.1 (±3.5)	62.5 (±3.3)	83.7 (±4.1)	68.3 (±3.7)	67.0 (±3.7)	89.5 (±0.9)	76.9 (±2.0)	70.2 (±3.2)
ergosta-7,22-dienol					0.7 (±0.6)				
14α-methylepisterol		0.4 (±0.8)	1.7 (±1.0)			0.4 (±0.8)			*
7-dehydrostigmasterol	17.2 (±2.5)	11.7 (±3.8)	0.8 (±1.3)	16.3 (±4.1)	18.3v (±1.1)	7.3 (±1.2)	10.5 (±0.9)	9.2 (±1.7)	7.3 (±2.9)
obtusifoliol + obtusifolione		3.5 (±1.9)	13.6 (±3.4)		1.7 (±0.8)	6.9 (±0.8)		2.5 (±0.7)	5.4 (±0.6)
eburicol		5.2 (±3.5)	21.5 (±4.3)		11.1 (±3.0)	18.5 (±1.4)		11.5 (±3.4)	17.0 (±4.2)

*: sterol detected 3 times (<1%).

**Table 6:** Relative composition of sterols of group 4, strains with TR_53_ alterations, under basal conditions or after in vitro exposure to itraconazole (ITC) (concentration = 32 mg/L except AF124: concentration = 0.25 × MIC) or voriconazole (VRC) (concentration = 0.25 × MIC). Values are expressed as the mean of three independent experiments, standard deviation is given in brackets.

	AF84			AF112			AF124		
	Basal	ITC	VRC	Basal	ITC	VRC	Basal	ITC	VRC
ergosterol Z et E	89.1 (±2.9)	67.2 (±3.6)	74.2 (±5.1)	85.9 (±4.1)	70.3 (±3.6)	53.7 (±3.9)	89.1 (±2.9)	61.8 (±12.8)	58.9 (±6.6)
ergosta-7,22-dienol		1.1 (±1.0)							
14α-methylepisterol		**	**			0.5 (±0.9)		2.5 (±2.4)	2.3 (±0.6)
Unknown 2								1.2 (±0.1)	
7-dehydrostigmasterol	10.9 (±2.9)	12.6 (±5.9)	8.6 (±4.9)	14.1 (±4.0)	15.7 (±3.1)	1.1 (±1.5)	10.9 (±2.9)	2.5 (±4.3)	1.5 (±0.1)
14α-methylfecosterol									***
obtusifoliol + obtusifolione		4.6 (±0.4)	5.6 (±0.5)		3.3 (±0.3)	13.0 (±1.2)		14.5 (±6.8)	15.2 (±2.8)
eburicol		14.5 (±1.5)	11.6 (±10.2)		10.7 (±2.1)	32.0 (±2.7)		17.7 (±8.9)	22.5 (±3.4)

**: sterol detected 2 times (<1%); ***: sterol detected 1 time (<1%).

**Table 7:** Comparison of basal relative (%) composition of sterols of six WT strains of *A. fumigatus* published between 2001 and 2019.

Reference	Isolates Name	Condition of Growth			Relative Composition of Sterols					
		Time	T °C	Liquid Medium	1	2	3	4	5	6 **
[12,16]	CM237	18 h	37 °C	MM	73.5–75.8	4.7–6.1	1.9–2.7	1.3–1.6	1.1	13.8–17.0
[13]	ATCC46645	Overnight	37 °C	RPMI 1640	95.0					5.0
[14]	ASFU1112	24 h	35 °C	Sabouraud	89.9	0.9	0.3			8.9
	ASFU1119				87.1		0.3			12.6
	ASFU1463 *				75.5	19.4				5.1
[19]	ATCC MYA-4609	48 h	37 °C	Sabouraud	90.8		0.6			8.6
This study		48 h	37 °C	YPD	79.5–89.5	10.5–20.0	<1.0	<1.0	<1.0	

1: Ergosterol; 2: 7-dehydrostigmasterol; 3: Eburicol; 4: 4-methylfecosterol; 5: 9-dehydroergosterol; 6: Other sterols. *: environmental isolate ** these sterols were not detected in our study under basal conditions; T °C: Temperature; MM: minimal medium; YPD: Yeast-extract Peptone Dextrose.

## Data Availability

The data presented in this study are available on request from the corresponding author.

## References

[1] Ullmann A.J., Aguado J.M., Arikan-Akdagli S., Denning D.W., Groll A.H., Lagrou K., Lass-Flörl C., Lewis R.E., Munoz P.E., Verweij P.E. (2018). Diagnosis and management of *Aspergillus* diseases: Executive summary of the 2017 ESCMID-ECMM-ERS guideline. Clin. Microbiol. Infect..

[2] Patterson T.F., Thompson G.R., Denning D., Fishman J.A., Hadley S., Herbrecht R., Kontoyiannis D.P., Marr K.A., Morrison V.A., Nguyen M.H. (2016). Executive Summary: Practice Guidelines for the Diagnosis and Management of Aspergillosis: 2016 Update by the Infectious Diseases Society of America. Clin. Infect. Dis..

[3] Arastehfar A., Lass-Flörl C., Garcia-Rubio R., Daneshnia F.F., Ilkit M., Boekhout T., Gabaldon T., Perlin D.S. (2020). The Quiet and Underappreciated Rise of Drug-Resistant Invasive Fungal Pathogens. J. Fungi.

[4] Alcazar-Fuoli L., Mellado E. (2013). Ergosterol biosynthesis in *Aspergillus fumigatus*: Its relevance as an antifungal target and role in antifungal drug resistance. Front. Microbiol..

[5] Verweij P.E., Mellado E., Melchers W. (2007). Multiple-Triazole–Resistant Aspergillosis. New Engl. J. Med..

[6] Mellado E., Garcia-Effron G., Alcázar-Fuoli L., Melchers W.J.G., Verweij P., Cuenca-Estrella M., Rodríguez-Tudela J.L. (2007). A New *Aspergillus fumigatus* Resistance Mechanism Conferring In Vitro Cross-Resistance to Azole Antifungals Involves a Combination of cyp51A Alterations. Antimicrob. Agents Chemother..

[7] Lestrade P.P.A., Meis J.F., Melchers W.J.G., Verweij P.E. (2019). Triazole resistance in *Aspergillus fumigatus*: Recent insights and challenges for patient management. Clin. Microbiol. Infect..

[8] Nywening A.V., Rybak J.M., Rogers P.D., Fortwendel J.R. (2020). Mechanisms of triazole resistance in *Aspergillus fumigatus*. Environ. Microbiol..

[9] Garcia-Rubio R., Cuenca-Estrella M., Mellado E. (2017). Triazole Resistance in *Aspergillus* Species: An Emerging Problem. Drugs.

[10] Martel C.M., Parker J.E., Bader O., Weig M., Gross U., Warrilow A.G.S., Kelly D.E., Kelly S.L. (2010). A Clinical Isolate of *Candida albicans* with Mutations in ERG11 (Encoding Sterol 14α-Demethylase) and ERG5 (Encoding C22 Desaturase) Is Cross Resistant to Azoles and Amphotericin B. Antimicrob. Agents Chemother..

[11] Ghannoum M.A., Spellberg B.J., Ibrahim A.S., Ritchie J.A., Currie B., Spitzer E.D., Edwards J.E., Casadevall A. (1994). Sterol composition of Cryptococcus neoformans in the presence and absence of fluconazole. Antimicrob. Agents Chemother..

[12] Alcazar-Fuoli L., Mellado E., Garcia-Effron G., Lopez J.F., Grimalt J.O., Cuenca-Estrella J.M., Rodriguez-Tudela J.L. (2008). Ergosterol biosynthesis pathway in *Aspergillus fumigatus*. Steroids.

[13] Müller C., Neugebauer T., Zill P., Lass-Flörl C., Bracher F., Binder U. (2018). Sterol Composition of Clinically Relevant Mucorales and Changes Resulting from Posaconazole Treatment. Mol..

[14] Dannaoui E., Persat F., Borel E., Piens M.A., Picot S. (2001). Sterol composition of itraconazole-resistant and itraconazole-susceptible isolates of *Aspergillus fumigatus*. Can. J. Microbiol..

[15] Hagiwara D., Arai T., Takahashi H., Kusuya Y., Watanabe A., Kamei K. (2018). Non-cyp51A Azole-Resistant *Aspergillus fumigatus* Isolates with Mutation in HMG-CoA Reductase. Emerg. Infect. Dis..

[16] Alcazar-Fuoli L., Mellado E., Garcia-Effron G., Buitrago M.J., López J., Grimalt J., Cuenca-Estrella J.M., Rodriguez-Tudela J.L. (2006). *Aspergillus fumigatus* C-5 Sterol Desaturases Erg3A and Erg3B: Role in Sterol Biosynthesis and Antifungal Drug Susceptibility. Antimicrob. Agents Chemother..

[17] Rybak J.M., Ge W., Wiederhold N.P., Parker J.E., Kelly S.L., Rogers P.D., Fortwendel J.R. (2019). Mutations in hmg1, Challenging the Paradigm of Clinical Triazole Resistance in *Aspergillus fumigatus*. mBio.

[18] Furukawa T., Van Rhijn N., Fraczek M., Gsaller F., Davies E., Carr P., Gago S., Fortune-Grant R., Rahman S., Gilsenan J.M. (2020). The negative cofactor 2 complex is a key regulator of drug resistance in *Aspergillus fumigatus*. Nat. Commun..

[19] Warrilow A., Parker J., Price C.L., Rolley N.J., Nes W.D., Kelly D.E., Kelly S.L. (2019). Isavuconazole and voriconazole inhibition of sterol 14α-demethylases (CYP51) from *Aspergillus fumigatus* and *Homo sapiens*. Int. J. Antimicrob. Agents.

[20] Arendrup M., Meletiadis J., Mouton J.W., Lagrou K., Hamal P., Guinea J. The Subcommittee on Antifungal Susceptibility Testing (AFST) of the ESCMID European Committee for Antimicrobial Susceptibility Testing. EUCAST Definitive Document E.Def 9.3.2: Method for the Determination of Broth Dilution Minimum Inhibitory Concentrations of Antifungal Agents for Conidia Forming Moulds. https://www.eucast.org/astoffungi/methodsinantifungalsusceptibilitytesting/ast_of_moulds/.

[21] Müller C., Junker J., Bracher F., Giera M. (2019). A gas chromatography–mass spectrometry-based whole-cell screening assay for target identification in distal cholesterol biosynthesis. Nat. Protoc..

[22] The Lipid Web. https://www.lipidmaps.org/resources/lipidweb/index.php?page=index.html.

[23] Akihisa T., Goad J. (1997). Analyse des Sterols.

[24] Müller C., Binder U., Bracher F., Giera M. (2017). Antifungal drug testing by combining minimal inhibitory concentration testing with target identification by gas chromatography–mass spectrometry. Nat. Protoc..

[25] Müller C., Staudacher V., Krauss J., Giera M., Bracher F. (2013). A convenient cellular assay for the identification of the molecular target of ergosterol biosynthesis inhibitors and quantification of their effects on total ergosterol biosynthesis. Steroids.

[26] Brooks C.J.W., Horning E.C., Young J.S. (1968). Characterization of sterols by gas chromatography-mass spectrometry of the trimethylsilyl ethers. Lipids.

[27] Weete J.D., Gandhi S.R. (1997). Sterols of the Phylum Zygomycota: Phylogenetic Implications. Lipids.

[28] Quail M.A., Arnold A., Moore D.J., Goosey M.W., Kelley S.L. (1993). Ketoconazole-mediated growth inhibition in *Botrytis cinerea* and *Saccharomyces cerevisiae*. Phytochemistry.

[29] Shirane N., Murabayashi A., Masuko M., Uomori A., Yoshimura Y., Seo S., Uchida K., Takeda K. (1990). Effect on ergosterol biosynthesis of a fungicide, SSF-109, in *Botrytis cinerea*. Phytochemistry.

[30] Moss G.P. (1989). Nomenclature of steroids (Recommendations 1989). Pure Appl. Chem..

[31] Bouvier-Nave P., Husselstein T., Benveniste P. (1998). Two families of sterol methyltransferases are involved in the first and the second methylation steps of plant sterol biosynthesis. JBIC J. Biol. Inorg. Chem..

[32] Nes W. (2000). Sterol methyl transferase: Enzymology and inhibition. Biochim. et Biophys. Acta BBA Bioenerg..

[33] Lepesheva G.I., Waterman M.R. (2011). Sterol 14alpha-demethylase (CYP51) as a therapeutic target for human trypanosomiasis and leishmaniasis. Curr. Top. Med. Chem..

[34] Nes W., Xu S., Haddon W.F. (1989). Evidence for similarities and differences in the biosynthesis of fungal sterols. Steroids.

[35] Lv Q.-Z., Qin Y.-L., Yan L., Wang L., Zhang C., Jiang Y.-Y. (2018). NSG2 (ORF19.273) Encoding Protein Controls Sensitivity of *Candida albicans* to Azoles through Regulating the Synthesis of C14-Methylated Sterols. Front. Microbiol..

[36] Georgopapadakou N.H., Walsh T.J. (1996). Antifungal agents: Chemotherapeutic targets and immunologic strategies. Antimicrob. Agents Chemother..

[37] Martel C.M., Parker J.E., Bader O., Weig M., Gross U., Warrilow A.G.S., Rolley N., Kelly D.E., Kelly S.L. (2010). Identification and Characterization of Four Azole-Resistant erg3 Mutants of *Candida albicans*. Antimicrob. Agents Chemother..

[38] Marichal P., Gorrens J., Laurijssens L., Vermuyten K., Van Hove C., Le Jeune L., Verhasselt P., Sanglard D., Borgers M., Ramaekers F.C.S. (1999). Accumulation of 3-Ketosteroids Induced by Itraconazole in Azole-Resistant Clinical *Candida albicans* Isolates. Antimicrob. Agents Chemother..

[39] Venkateswarlu K., Kelly S.L. (1996). Biochemical characterisation of ketoconazole inhibitory action on *Aspergillus fumigatus*. FEMS Immunol. Med Microbiol..

[40] Sherald J.L., Sisler H.D. (1975). Antifungal mode of action of triforine. Pestic. Biochem. Physiol..

[41] Nes W.D. (2011). Biosynthesis of Cholesterol and Other Sterols. Chem. Rev..

[42] Snelders E., Karawajczyk A., Verhoeven R.J., Venselaar H., Schaftenaar G., Verweij P.E., Melchers W.J. (2011). The structure–function relationship of the *Aspergillus fumigatus* cyp51A L98H conversion by site-directed mutagenesis: The mechanism of L98H azole resistance. Fungal Genet. Biol..

[43] Gsaller F., Hortschansky P., Furukawa T., Carr P.D., Rash B., Capilla J., Muller C., Bracher F., Bowyer P., Haas H. (2016). Sterol Biosynthesis and Azole Tolerance Is Governed by the Opposing Actions of SrbA and the CCAAT Binding Complex. PLoS Pathog..

[44] Snelders E., Camps S.M., Karawajczyk A., Rijs A.J., Zoll J., Verweij P., Melchers W.J. (2015). Genotype–phenotype complexity of the TR46/Y121F/T289A cyp51A azole resistance mechanism in *Aspergillus fumigatus*. Fungal Genet. Biol..

[45] Watson P., Rose M., Ellis S., England H., Kelly S. (1989). Defective sterol C5-6 desaturation and azole resistance: A new hypothesis for the mode of action of azole antifungals. Biochem. Biophys. Res. Commun..

[46] Kelly S.L., Lamb D.C., Kelly D.E., Manning N.J., Loeffler J., Hebart H., Schumacher U., Einsele H. (1997). Resistance to fluconazole and cross-resistance to amphotericin B in *Candida albicans* from AIDS patients caused by defective sterol Δ5,6 -desaturation. FEBS Lett..

